# Ulcerative Colitis and Perfluorooctanoic Acid (PFOA) in a Highly Exposed Population of Community Residents and Workers in the Mid-Ohio Valley

**DOI:** 10.1289/ehp.1206449

**Published:** 2013-06-04

**Authors:** Kyle Steenland, Liping Zhao, Andrea Winquist, Christine Parks

**Affiliations:** 1 Rollins School of Public Health, Emory University, Atlanta, Georgia, USA; 2 National Institute for Environmental Health Sciences, National Institutes of Health, Department of Health and Human Services, Research Triangle Park, North Carolina, USA

**Keywords:** autoimmune, inflammatory bowel disease, PFOA, ulcerative colitis

## Abstract

Background: Little is known about environmental determinants of autoimmune diseases.

Objectives: We studied autoimmune diseases in relation to level of exposure to perfluorooctanoic acid (PFOA), which was introduced in the late 1940s and is now ubiquitous in the serum of residents of industrialized countries.

Methods: In 2008–2011 we interviewed 32,254 U.S. adults with high serum PFOA serum levels (median, 28 ng/mL) associated with drinking contaminated water near a chemical plant. Disease history was assessed retrospectively from 1952 or birth (if later than 1952) until interview. Self-reported history of autoimmune disease was validated via medical records. Cumulative exposure to PFOA was derived from estimates of annual mean serum PFOA levels during follow-up, which were based on plant emissions, residential and work history, and a fate-transport model. Cox regression models were used to estimate associations between quartiles of cumulative PFOA serum levels and the incidence of autoimmune diseases with ≥ 50 validated cases, including ulcerative colitis (*n* = 151), Crohn’s disease (*n* = 96), rheumatoid arthritis (*n* = 346), insulin-dependent diabetes (presumed to be type 1) (*n* = 160), lupus (*n* = 75), and multiple sclerosis (*n* = 98).

Results: The incidence of ulcerative colitis was significantly increased in association with PFOA exposure, with adjusted rate ratios by quartile of exposure of 1.00 (referent), 1.76 (95% CI: 1.04, 2.99), 2.63 (95% CI: 1.56, 4.43), and 2.86 (95% CI: 1.65, 4.96) (*p*_trend_ < 0.0001). A prospective analysis of ulcerative colitis diagnosed after the baseline 2005–2006 survey (*n* = 29 cases) suggested a positive but non-monotonic trend (*p*_trend_ = 0.21).

Discussion: To our knowledge, this is the first study of associations between this common environmental exposure and autoimmune diseases in humans. We found evidence that PFOA is associated with ulcerative colitis.

## Introduction

Autoimmune diseases affect about 7% of the U.S. population (Cooper et al. 2009; Miller et al. 2012). The most common autoimmune diseases include inflammatory bowel disease (consisting of ulcerative colitis and Crohn’s disease), rheumatoid arthritis, celiac disease, multiple sclerosis, systemic sclerosis, systemic lupus, type 1 diabetes, and two types of thyroid disease (Grave’s disease and Hashimoto’s thyroiditis) (Cooper et al. 2009). In general, these diseases are chronic and incurable. They are thought to occur as a result of an interaction between genes and the environment. Twin studies on concordance suggest a relatively low genetic contribution for rheumatoid arthritis, ulcerative colitis, and systemic sclerosis; a modest contribution for Crohn’s disease and lupus; and a high contribution for type 1 diabetes and autoimmune thyroid disease. Data are largely lacking for other autoimmune diseases (Baumgart and Carding 2007; Bogdanos et al. 2012). Genetic studies, including recent genomewide association studies (GWAS), have identified a number of genes that may contribute to autoimmune diseases, particularly genes of the major histocompatibility complex (MHC) that have associated with rheumatoid arthritis, systemic sclerosis, and lupus (Delgado-Vega et al. 2010). A number of specific genes have been associated with more than one autoimmune disease.

To date few environmental risk factors for autoimmune disease have been identified. The principal ones are *a*) crystalline silica, which has been associated with rheumatoid arthritis, systemic sclerosis, and lupus; *b*) smoking, positively associated with seropositive rheumatoid arthritis and negatively associated with ulcerative colitis; *c*) solvents, associated with systemic sclerosis; and *d*) sunlight, negatively associated with multiple sclerosis (Miller et al. 2012). Although information on population trends in autoimmune disease rates is limited, concerns have been raised that autoimmune disease incidence rates may be increasing as a result of increasing exposures to xenobiotics (Miller et al. 2012). One such xenobiotic, which is ubiquitous in the serum of U.S. residents, and which has been shown to affect immune responses in rodents (DeWitt et al. 2009), is perfluorooctanoic acid (PFOA). To our knowledge, there are no prior studies of associations between PFOA and autoimmune disease in humans.

In the present study, we focused on a large cohort (*n* = 32,254) that had been exposed to high levels of PFOA, a perfluorinated compound introduced into the environment in the 1950s. PFOA (also known as C8) is ubiquitous at low levels (nanograms per milliliter) in the serum of virtually all residents of industrialized countries (Lindstrom et al. 2011). Human exposure to PFOA occurs via many sources, including food, drinking water (Shin et al. 2011a, 2011b), house dust (Strynar and Lindstrom 2008), and air (Fromme et al. 2009). Until recently, PFOA has been used in manufacturing a wide variety of consumer products, such as Gore-tex™, Teflon®, and Scotchgard™ (Shin et al. 2011b). The cohort studied here was exposed primarily via drinking water contaminated with PFOA that originated from emissions from a nearby chemical plant that used PFOA in the manufacture of Teflon®. Median PFOA serum levels in this population in 2005–2006 were 28 ng/mL, and mean levels were 72 ng/mL, compared with an estimated mean serum concentration of 4 ng/mL for the general U.S. population (Calafat et al. 2007; Steenland et al. 2009).

PFOA is not biodegradable and persists indefinitely in the environment. In humans it is thought to be stored in the liver, kidney, and serum, and has an elimination half-life of approximately 3.5 years (Olsen et al. 2007). PFOA is a rodent carcinogen and causes fetal loss in mice at dietary levels of approximately 20 ppm (Lau et al. 2007). Rodent studies have reported evidence of immunosuppressant effects including lymphoid organ atrophy and decreased de novo antibody production in certain strains of mice (DeWitt et al. 2008; Yang et al. 2000). To date, information about PFOA in relation to immune function in humans is limited to a few cross-sectional studies of relatively insensitive immune markers (reviewed by Steenland et al. 2010). No clear patterns of immune suppression have been reported based on these studies. However, a recent study of children in the Faroe Islands reported a significant decrease in childhood antibody levels to diphtheria and tetanus at 7 years of age in association with elevated serum levels of PFOA and another fluorocarbon, perfluoroctane sulfonic acid (PFOS) (Grandjean et al. 2012). To our knowledge, there are no published data on autoimmune disease in relation to PFOA in animals or humans.

The autoimmune diseases considered here are ulcerative colitis, Crohn’s disease, rheumatoid arthritis, multiple sclerosis, lupus, and type 1 diabetes. For these six diseases we had data on self-reported occurrence, for which we then sought validation through medical records review. Analyses here are limited to cases validated based on medical records data. For most other autoimmune diseases we did not have self-reported data, or the numbers of cases were too small to analyze. Self-reported cases of Graves’ disease and Hashimoto’s thyroiditis are not included in this analysis because they were difficult to distinguish from other (non-autoimmune) forms of hyperthyroidism and hypothyroidism.

Although it is likely that different autoimmune diseases have separate etiologies, the fact that several genes have been linked to more than one autoimmune disease also suggests that they may have some aspects in common (Delgado-Vega et al. 2010). Here we have analyzed the relationship of each autoimmune disease to PFOA exposure.

This study is one of a series of studies conducted by the C8 Science Panel, a three-person panel of epidemiologists set up pursuant to a 2004 legal settlement between plaintiffs and DuPont (C8 Science Panel 2013; Frisbee et al. 2009). The settlement mandated a baseline survey of 69,000 persons who lived or who had lived in six water districts where water had been contaminated with PFOA (called the C8 Health Project, or C8HP). The settlement also created the C8 Science Panel, which was charged with determining whether it is more probable than not that PFOA is linked to (associated with) any human disease. The C8 Science Panel conducted 11 studies over the course of 5 years to make this determination.

## Methods

*Study population and exposure estimation*. Community cohort. We studied a Mid-Ohio Valley community-based population that comprised persons who lived or worked in any of six PFOA-contaminated water districts and participated in the C8HP baseline survey in 2005–2006 (Frisbee et al. 2009). The principal route of exposure for this population was via PFOA-contaminated drinking water. C8HP participants (*n* = 69,030) had their PFOA serum levels measured and provided a medical history. Approximately 80% of the current residents who had participated in the 2005–2006 survey in the six districts participated in the C8HP (Frisbee et al. 2009).

Most adult C8HP participants (74% of participants ≥ 20 years of age) consented to participate in a follow-up study. Of these we subsequently interviewed 82% (*n* = 32,712). These interviews form the basis for the present study. For further details of the interview process, see Winquist et al. (2013).

Two rounds of surveys were conducted, the first from August 2008 to April 2010 and the second from May 2010 to May 2011. Surveys were completed over the phone (63%) or online (37%), and most participants completed both rounds of surveys. Both surveys included the same questions regarding demographic information and medical history, including questions about whether the participants had ever been told by a doctor that they had specific chronic diseases. For participants who died after the completion of the C8HP survey in 2005–2006 or who were not capable of completing a follow-up survey, we surveyed their next-of-kin (4% of the cohort had next-of-kin interview). The Emory University Institutional Review Board reviewed and approved all aspects of this study, including consent forms and surveys.

To estimate past exposures, we developed historical yearly serum PFOA estimates for community residents based on the estimated intake of PFOA-contaminated drinking water, assuming low background exposure. The estimates of drinking-water PFOA concentrations were based on the amount of PFOA released from the DuPont plant, wind patterns, river flow, groundwater flow, and the residential address history provided by each participant (Shin et al. 2011a, 2011b). Each estimated yearly serum concentration took into account new exposure during the year and the estimated amount of PFOA remaining in the body after partial excretion during the prior year. The final community cohort comprised 28,541 community residents who had estimated historical PFOA serum concentrations and had never worked at the DuPont plant.

Worker cohort. In addition to the community cohort, we studied a cohort of 4,391 past and current workers at the DuPont plant using the same survey completed by the community cohort participants, including a residential history. This group was a subset of a cohort of 6,027 workers employed at the DuPont chemical plant between 1948 and 2002 who were previously studied for mortality (Leonard et al. 2008; Steenland and Woskie 2012).

Estimates of annual serum PFOA levels for workers in different jobs were developed by the C8 Science Panel (Woskie et al. 2012) and combined with estimated annual serum levels from residential exposure to contaminated drinking water that were derived as described above for the community cohort. We estimated combined residential and occupational exposure for 3,713 workers who completed a follow-up survey (84%), including 1,890 (51%) who also had participated in the 2005–2006 C8HP.

Combined community and worker population. Community residents and workers were combined to form a final population of 32,254 persons for whom we could study the relationship between past PFOA serum levels and subsequent disease. Our model-based exposure predictions were well correlated with serum PFOA measurements obtained in 2005–2006 for community cohort members (*r* = 0.67) (Shin et al. 2011b).

*Medical history*. All participants were asked about lifetime medical history, focusing on chronic disease. Participants who reported that they had been told they had one of the specific chronic diseases of interest were then asked the age at first diagnosis and for consent to review medical records as well as for consent to contact the relevant health care provider. Approximately 60% of the cohort reported having had a disease of interest; 79% percent of these consented for us to review their medical history. Professional medical abstractors obtained records documenting the disease in question from medical providers by mail or by in-person office visit. Overall, we were able to find a medical record relevant to the reported disease for 92% of those consenting to medical record review (Winquist et al. 2013). For three diseases—multiple sclerosis, rheumatoid arthritis, and lupus—we were also able to use supplemental data from the C8HP (2005–2006), which conducted its own medical record validation, to confirm some cases that had been self-reported to us but for whom were unable to obtain a medical record.

*Autoimmune disease in the cohort*. Participants were asked whether a doctor or other health professional had ever told them they had an autoimmune disease, with specific categories listed for lupus, multiple sclerosis, myasthenia gravis, Sjögren’s syndrome, vasculitis, Addison’s disease, or other. If the answer was “other,” the participant was asked to specify the disease. In a separate question, participants were asked whether a doctor or health professional had ever told them they had had inflammatory bowel disease, excluding irritable bowel syndrome. They were then asked to specify whether the inflammatory bowel disease was ulcerative colitis or Crohn’s disease. In another question, participants were asked whether a doctor had ever told them they had arthritis, and if yes, whether it was rheumatoid or osteoarthritis. Finally, participants were asked if they had ever had diabetes, with specific categories of type 1 or type 2 (excluding pregnancy-induced diabetes).

*Analyses*. Associations between PFOA exposure and each outcome were conducted using separate survival analyses (Cox regression) with age as the time variable. Follow-up began in 1952 (the approximate date of first emissions of PFOA from the DuPont plant), or date of birth, whichever came later. Years outside the study area were included in follow-up and assigned U.S. background levels, although sensitivity analyses were conducted excluding person-time prior to arrival in the Mid-Ohio Valley and/or excluding background levels in calculating cumulative exposure. For each outcome, follow-up ended at time of last interview, at time of disease occurrence, or at time of death, whichever occurred earliest. Cases of each outcome were restricted to cases validated based on medical record review. Participants who self-reported an outcome that was not validated were excluded from the analysis for that outcome.

We estimated associations with cumulative exposure to PFOA, which was calculated by summing estimated yearly serum concentrations during follow-up and modeled as a time-dependent variable in Cox regression. We also considered cumulative exposure with a lag of 10 years (i.e., discounting exposure in the prior 10 years). We conducted analyses by quartile of cumulative exposure, with cut points for each outcome determined from the cumulative exposure of the cases of that outcome at time of diagnosis, and separate cut points derived for lagged and unlagged exposures. Rate ratios (RR; the ratio of disease rates) were estimated for the second, third, and fourth quartiles relative to the first quartile, which was the referent group. Tests for trend were based on the *p*-value of the coefficient of the natural log of cumulative exposure modeled as a continuous time-dependent variable in a Cox regression model that included the same covariates as the corresponding model of cumulative PFOA exposure as a categorical variable.

All models were adjusted for a standard set of covariates including sex and race/ethnicity (white or non-white) and time-dependent variables for smoking (current, former, vs. never), BMI (body mass index) (< 18.5, 18.5–24.9, 25–29.9, ≥ 30.0 kg/m^2^), and alcohol consumption (current, former, vs. never) based on the most recent survey. The proportional hazard assumption of a constant hazard ratio for cumulative exposure over time was verified based on the nonsignificance of an interaction term between time (age) and cumulative exposure.

We conducted two types of sensitivity analyses. In one (qualifying time analysis), the time of follow-up started when the participant qualified for the C8HP (i.e., time of first residence in a PFOA-contaminated water district or first employment at the plant). This eliminated prior nonexposed person-time before cohort eligibility, and resulted in a loss of about 10–15% of cases, depending on the outcome being evaluated. Second, we estimated associations with cumulative exposures above background levels only, so that exposure began at year of first exposure to contaminated water or work in the DuPont plant (above background analysis). These analyses included the same number of participants for each outcome as corresponding models of associations with cumulative PFOA exposure including estimated background levels of exposure (approximately 4 ng/mL serum).

Other analyses were restricted to person-time after the C8HP was established in 2005–2006 (prospective analysis), with participants reporting a history of any of the autoimmune diseases before participation in the C8HP (prevalent cases) being excluded to restrict the analysis to incident cases diagnosed after 2005–2006. Person-time for participants in the C8HP started on the date of their C8HP blood draw and interview, which varied from July 2005 to July 2006. For members of the worker cohort who did not participate in the C8HP (*n* = 1,823), person-time at risk began on 1 July 2006. For all but two of the autoimmune diseases—rheumatoid arthritis and ulcerative colitis—there were insufficient cases to conduct a prospective analysis.

## Results

Table 1 provides descriptive statistics for the cohort. The median year of birth of the cohort was 1957, 54% were female, 20% were current smokers, 27% were current drinkers, 18% had a college education or more, and 36% were obese (BMI > 30) (BMI, smoking, and drinking assessed at time of last interview). PFOA serum levels in 2005–2006 were 24 ng/m^3^ for community residents and 113 ng/m^3^ for workers. The median length of follow up was 53 years (birth to end of follow-up); the median length of follow-up after the earliest year living or working in a contaminated water district was 29 years.

**Table 1:** Cohort characteristics based on the most recent survey and measured serum PFOA levels in 2005–2006 [*n* (%)].

Characteristic	Community cohort (*n *= 28,541)	Worker cohort (*n *= 3,713)	Combined cohorts (*n *= 32,254)
Year of birth
25th percentile	1947	1941	1946
Median	1958	1951	1957
75th percentile	1970	1963	1969
Sex
Female	16,602 (58)	758 (20)	17,360 (54)
Race/ethnicity
White, non-Hispanic	27,901 (98)	3,284 (88)	31,185 (97)
Other	640 (2)	134 (4)	774 (2)
Missing	0	295 (8)	295 (1)
BMI (kg/m³)
< 18.5	414 (2)	38 (1)	452 (1)
18.5–< 25.0	7,693 (27)	972 (26)	8,665 (27)
25.0–< 30.0	9,689 (34)	1,618 (44)	11,307 (35)
≥ 30.0	10,694 (37)	1,057 (29)	11,751 (37)
Education
< High school	3,026 (11)	37 (1)	3,063 (10)
High school	11,706 (41)	1,265 (34)	12,971 (40)
Some college	9,441 (33)	1,081 (29)	10,522 (33)
≥ College diploma	4,366 (15)	1,328 (36)	5,694 (18)
Missing	2 (< 1)	2 (< 1)	4 (< 1)
Smoking
Never smoked	13,527 (47)	1,989 (54)	15,516 (48)
Smoked and quit	8,899 (31)	1,297 (35)	10,196 (32)
Smoked, did not quit	6,115 (21)	427 (12)	6,542 (20)
Regular alcohol consumption
Never	17,011 (60)	1,683 (45)	18,694 (58)
Yes and quit	4,105 (14)	535 (14)	4,640 (14)
Yes, did not quit	7,360 (26)	1,486 (40)	8,846 (27)
In community cohort	28,541 (100)	1,890 (51)	30,431 (94)
Serum PFOA concentration measurement from the C8HP (2005–2006) (ng/mL)^*a*^			
Mean	71	325	87
SD	151	921	278
Interquartile range	12–59	56–256	13–68
Median	24	113	26
^***a***^Serum PFOA data for members of the worker cohort are restricted to 1,890 workers who had serum PFOA measurements because of participation in the C8HP in 2005–2006. All other data for workers are from survey data and refer to all 3,713 workers.			

Numbers of validated cases of each autoimmune disease are shown along with mean at diagnosis and sex in Table 2. Validated cases (based on medical record review) included 346 participants who reported rheumatoid arthritis for which they were taking medication (25% of the 1,292 who reported taking medication), 72 participants with lupus (39% of 187 self-reported cases), and 98 with multiple sclerosis (65% of 150 self-reported cases). There were 151 validated cases of ulcerative colitis (25% of self-reported cases), and 96 validated cases of Crohn’s disease (53% of self-reported cases) (10 cases were validated for both ulcerative colitis and Crohn’s disease).

**Table 2:** Numbers of participants with validated auto­immune diseases, mean age at diagnosis, and sex.^*a*^

Autoimmune disease	No. self-reported^*b*^	No. validated^*b*^	Mean age	Percent female^*c*^
Ulcerative colitis	596	151	44	59
Crohn’s disease	178	95	39	59
Rheumatoid arthritis	1,292	346	48	69
Type 1 diabetes–broad^*d*^	342	160	35	52
Type 1 diabetes–narrow^*e*^	NA	85	27	48
Lupus	187	72	43	87
Multiple sclerosis	150	99	38	77
NA, not applicable. ^***a***^Cases confirmed through medical records review. ^***b***^In the entire cohort, among people reporting diseases for which validation was sought, ~ 75% consented to medical record review, and among those who consented, a record was obtained for 92%; among these the percent validated across all diseases was 77%. For multiple sclerosis, rheuma­toid arthritis, and lupus, we included among the validated those cases which had been self-reported and not confirmed by us for lack of a medical record, but which had been confirmed previously during the C8HP (29, 19, and 5 cases respectively). For rheuma­toid arthritis, numbers are restricted to cases who reported taking medication; these were the only ones for which we sought medical records. ^***c***^54% of the entire cohort was female. ^***d***^Includes cases self-reported as type 1 diabetes and then verified as either type 1 diabetes or insulin-dependent diabetes. ^***e***^Restricted to the same self-reported cases specifically validated for type 1 diabetes.				

Of the 342 self-reported cases of type 1 diabetes, 69 were validated as either type 1 diabetes or insulin-dependent diabetes (type 1 diabetes is also known as insulin-dependent diabetes), and we found another 91 validated cases (as either insulin-dependent or type 1) among subjects who reported that they had diabetes but did not know what kind. Of the 160 validated cases, 85 were validated specifically for type 1 diabetes, while the remaining cases were validated as “insulin-dependent.” We conducted analyses for both the group of 160 validated cases and also for the subgroup of 85 cases specifically validated as type 1 diabetes.

The known higher incidence of rheumatoid arthritis, lupus, and multiple sclerosis among women compared with men is reflected in our data (Table 2). The age at diagnoses also tend to conform to what is known, e.g., most of these diseases tend to occur during middle age. The mean age for type 1 diabetes (combining insulin-dependent and specifically type 1) is younger than the mean age at onset for the other diseases, reflecting the fact that some of these cases occur among juveniles, although in recent years the majority of type 1 diabetes now occurs among adults.

Table 3 shows the results of the Cox regression for the overall analysis. Ulcerative colitis showed a significant positive association with cumulative PFOA exposure, with monotonically increasing rate ratios for both the unlagged and lagged exposures and significant trends based on models of log-transformed cumulative exposure. No such trend was evident for Crohn’s disease or for any of the other autoimmune diseases examined.

**Table 3:** Overall retrospective survival analysis results based on follow-up from 1952 through 2008–2011 [RR (95%)].^*a*^

	Q2 vs. Q1^*b*^	Q3 vs. Q1	Q4 vs. Q1	*p*_trend_ of log cumulative exposure
Unlagged exposure
Ulcerative colitis	1.76 (1.04, 2.99)	2.63 (1.56, 4.43)	2.86 (1.65, 4.96)	< 0.0001
Crohn’s disease	1.25 (0.61, 2.58)	1.15 (0.55, 2.41)	1.00 (0.48, 2.09)	0.73
Rheumatoid arthritis	1.24 (0.85, 1.79)	1.40 (0.96, 2.03)	0.99 (0.68, 1.43)	0.84
Type 1 diabetes–broad^*c*^	0.68 (0.29, 1.58)	0.53 (0.22, 1.30)	0.54 (0.22, 1.33)	0.84
Type 1 diabetes–narrow^*d*^	0.83 (0.25, 2.78)	1.41 (0.40, 4.95)	0.88 (0.25, 3.06)	0.68
Lupus	1.49 (0.68, 3.34)	1.01 (0.44, 2.30)	0.71 (0.31, 1.65)	0.94
Multiple sclerosis	0.85 (0.44, 1.63)	1.56 (0.81, 3.00)	1.26 (0.65, 2.42)	0.22
10-year lagged exposure
Ulcerative colitis	1.71 (0.89, 3.27)	2.05 (1.07, 3.91)	3.05 (1.56, 5.96)	< 0.0001
Crohn’s disease	0.80 (0.32,1.99)	0.97 (0.36, 2.60)	0.69 (0.26, 1.82)	0.79
Rheumatoid arthritis	1.53 (0.61, 2.58)	1.73 (1.10, 2.71)	1.35 (0.87, 2.11)	0.73
Type 1 diabetes–broad^*c*^	0.42 (0.09, 2.00)	0.70 (0.14, 0.35)	0.38 (0.08, 1.93)	0.84
Type 1 diabetes–narrow^*d*^	0.50 (0.05, 4.90)	1.32 (0.14, 12.40)	0.71 (0.07, 7.14)	0.65
Lupus	0.79 (0.27, 2.34)	1.26 (0.40, 4.03)	0.61 (0.19, 1.91)	0.93
Multiple sclerosis	1.16 (0.54, 2.47)	1.62 (0.75, 3.52)	1.32 (0.61, 2.84)	0.59
Q, quartile. ^***a***^Follow up continued until the date of the last survey, the date of diagnosis for each individual outcome being evaluated, or on the date of death, whichever occurred first. ^***b***^Cut points for these quartiles varied by disease analyzed, as they were based on dividing the cases equally by quartiles. For example for ulcerative colitis, the cut points were 158, 586, and 3,500 ng/mL/year, which for a 50-year-old participant would correspond to average exposure of approximately < 3, 3–11, 11–70, and > 70 ng/mL/ year. ^***c***^Includes self-reported cases listed as verified as either type 1 diabetes or insulin-dependent diabetes. ^***d***^Restricted to those self-reported cases specifically validated for type 1 diabetes.				

Sensitivity analyses using either exposures beginning in the “qualifying year” or without background added in, were similar to those shown in Table 3 (data not shown). For ulcerative colitis, with no background exposures added, the quartile RRs were 1.00 (referent), 1.27 (95% CI: 0.78, 2.08), 2.08 (95% CI: 1.26, 3.44), and 2.30 (95% CI: 1.36, 3.91) (*p*_trend_ < 0.0001). The corresponding RRs with estimated person-time before moving to an exposed water district were 1.59 (95% CI: 0.95, 2.65), 2.40 (95% CI: 1.44, 3.99), and 2.33 (95% CI: 1.37, 3.97) (*p*_trend_ < 0.0001). Again, ulcerative colitis was the only outcome associated with PFOA.

Only ulcerative colitis (*n* = 30) and rheumatoid arthritis (*n* = 56) had ≥ 25 cases diagnosed after participation in the original C8HP (2005–2006) or 1 July 2006 (for those enrolled in the worker cohort who did not participate in the C8HP). All RRs for ulcerative colitis were elevated after the first quartile, but without a monotonic trend, based on this prospective analysis (*p*_trend_ = 0.21, unlagged analysis) (Table 4).

**Table 4:** Prospective survival analysis results, follow-up 2005–2006 through 2008–2011, for selected outcomes (≥ 30 cases) [RR (95%)].

	Q2 vs. Q1	Q3 vs. Q1	Q4 vs. Q1	*p*_trend_ of log cumulative exposure
Unlagged exposure
Ulcerative colitis	1.68 (0.62, 4.66)	2.10 (0.75, 5.85)	1.62 (0.57, 4.61)	0.21
Rheumatoid arthritis	0.81 (0.39, 1.68)	2.07 (1.00, 4.27)	0.52 (0.25, 1.09)	0.89
10-year lagged exposure
Ulcerative colitis	1.21 (0.43, 3.34)	2.16 (0.80, 5.81)	1.51 (0.43, 4.30)	0.12
Rheumatoid arthritis	0.31 (0.14, 0.71)	0.90 (0.41, 2.00)	0.32 (0.14, 0.72)	0.89
Q, quartile.				

## Discussion

For all six autoimmune diseases considered here, the prevalence in the Mid-Ohio Valley conformed broadly with that expected from the U.S. and Western European populations, although expected prevalences for some of these diseases are uncertain (Cooper et al. 2009). Crude prevalences for validated cases per 100,000 adults (≥ 20 years of age) in our cohort in 2008–2001 were 410, 300, 1,080, 500, 230, and 310 for ulcerative colitis, Crohn’s disease, rheumatoid arthritis, type 1 diabetes (including insulin-dependent), lupus, and multiple sclerosis, respectively. Data from the United States and Western Europe based on both hospital and non-hospital data suggest comparable patterns but lower prevalences for adults and children combined, with ranges of 140–300, 100–200, 310–830, 340–570, 30–150, and 50–360, respectively, for these same diseases (Cooper et al. 2009). On the other hand, a recent study based on medical claims data (*n* = 12 million) reported prevalences of 263 and 241 per 100,000 U.S. adults for ulcerative colitis and Crohn’s disease, respectively, which suggests that ulcerative colitis may be in excess in our population (Kappelman et al. 2013). These data also indicate that the prevalences of both types of adult inflammatory bowel disease have been slightly increasing over the 6-year period studied (2004–2009).

Cumulative PFOA exposures were associated with ulcerative colitis, but not Crohn’s disease, in our study population. Ulcerative colitis and Crohn’s disease are clinically distinct conditions, with distinguishing clinical, anatomical, and histological findings. Crohn’s disease can occur in any part of the gastrointestinal tract, although most cases start in the end of the small bowel. Ulcerative colitis is restricted to the colon and the rectum. Ulcerative colitis affects the mucosa (epithelial lining of the gut), whereas Crohn’s disease affects the whole bowel wall. However, there is no definitive diagnostic method to distinguish between them in about 10% of cases (Hanauer 2006; Sands 2004).

Smoking is positively associated with Crohn’s disease (Mahid et al. 2006). In contrast, current smoking is negatively associated with ulcerative colitis, though former smoking is a risk factor. A meta-analysis for smoking and ulcerative colitis found an RR = 0.58 for current smoking, and RR = 1.76 for former smoking (Mahid et al. 2006). It is reassuring that we found some support for these patterns in our data. Specifically, RRs for current and former smoking and ulcerative colitis were 0.63 (95% CI: 0.39, 1.01; *p* = 0.05) and 1.90 (95% CI 1.29, 2.82, *p* = 0.001), respectively. The RRs for current and former smokers for Crohn’s disease were 1.26 (95% CI: 0.28, 2.03; *p* = 0.35) and 0.94 (95% CI: 0.50, 1.76; *p* = 0.85), respectively.

Both ulcerative colitis and Crohn’s disease have a genetic component, but twin studies suggest this component is relatively modest for ulcerative colitis. The concordance of ulcerative colitis in monozygotic twins is 10%, and 3% in dizygotic twins, compared with 37% and 7%, respectively, for Crohn’s disease (Baumgart and Carding 2007). Ordas et al. (2012) have recently reviewed environmental risk factors for ulcerative colitis. Positive risk factors include prior gastrointestinal infections and oral contraceptive use, while protective factors include appendectomy and breastfeeding. We do not have data to control for these factors in our analysis. However, with regard to oral contraceptives and breastfeeding, we analyzed our data separately for men and women, and found little difference between these analyses (data not shown).

A growing body of evidence suggests inflammatory bowel disease may be due, in part, to deficiencies in innate immunity and an impaired intestinal epithelial barrier, resulting in damaging inflammatory responses to the microbial environment of the gastrointestinal tract (Baumgart and Carding 2007; Gersemann et al. 2012; Ordas et al. 2012). Animal models and human studies suggest that PFOA toxicity includes systemic suppression of adaptive immunity and antibody production (DeWitt et al. 2012; Grandjean et al. 2012, and the alteration of inflammatory pathways (De Witt et al. 2012). Many PFOA-related immune effects may be mediated through activation of peroxisome proliferator–activated receptors (PPAR)-α, PPAR-γ, or other receptors expressed in the colon, but non–receptor-mediated changes may also occur (DeWitt et al. 2012). Although PFOA-related activation of human PPAR-γ has not been established (Takacs and Abbott 2007; Vanden Heuvel et al. 2006), activation of PPAR-γ through endogenous and exogenous ligands appears to have antiinflammatory effects in colitis models and is being explored for therapeutic potential (Dubuquoy et al. 2006). These findings seem to contradict our finding of a positive association between PFOA and ulcerative colitis. However, specific gastrointestinal and local immune effects of PFOA are not described in the literature.

Plausible mechanisms linking PFOA and ulcerative colitis may include shifts in the balance of tissue macrophages towards an antiinflammatory M2 phenotype and/or a T helper (TH)_2_-like response to specific antigens (DeWitt et al. 2012). Experimental findings also suggest PPAR-γ activation may lead to reprogramming of tissue macrophages towards an M2 antiinflammatory phenotype, which may contribute to decreased vaccine efficacy or immunosuppression in diseases dependent on cytotoxic T-cell responses (DeWitt et al. 2012). Other studies suggest PFOA may induce shifts in the TH_1_/TH_2_ balance, increasing production of TH_2_-like cytokines involved in hypersensitivity responses (DeWitt et al. 2012). One TH_2_-type cytokine [interleukin (IL)-13] is thought to play a unique and critical role in ulcerative colitis in gut mucosal inflammatory response (Mannon and Reinisch 2012); however, PFOA-related effects on IL-13 expression have not been described. Ultimately, the context of the microbial environment may be especially relevant in explaining PFOA-related effects on ulcerative colitis. One study’s findings that PFOA fed to mice (0.02% of total diet by weight) leads to both systemic neutropenia and increased lipopolysaccharide-related cytokine release in other cell populations (e.g., macrophages) (Qazi et al. 2009), suggests a general increase in host susceptibility to infections (DeWitt et al. 2012) Together, these findings suggest that the association between PFOA and ulcerative colitis in the present study (in contrast with a lack of clear associations with Crohn’s or any of the other autoimmune diseases examined), might be explained by effects of PFOA on responses to bacterial exposures and other unique aspects of lower gastrointestinal toxicity that may not be reflected by systemic or other organ-specific immune effects.

It is possible that increased gut absorption of PFOA in subjects with ulcerative colitis (including possibly early still undiagnosed ulcerative colitis) could lead to higher measured serum PFOA levels, which could create a false appearance of a causal association, if measured levels were used to predict current (or even subsequent) ulcerative colitis. However, in our data we estimated associations with cumulative PFOA exposure predicted by a model that did not incorporate measured PFOA concentrations, hence “reverse causality” (by which disease might increase measured serum levels rather than serum levels preceding disease) is not relevant here.

There are a number of limitations to our analysis. Our cohort was largely a survivor cohort, with community members having to have been alive in 2005–2006 at the time of a baseline survey. If community residents with autoimmune disease were less likely to have survived until 2005–2006, they would have been less likely than other residents to have enrolled in the study, possibly biasing our results. However, two factors argue against this as an explanation for our positive findings for ulcerative colitis. First, life expectancy for those with ulcerative colitis is about the same as the general population (Davoli et al. 1997; Winther et al. 2003). Second, our prospective analyses of ulcerative colitis, although limited by small numbers, would be unaffected by this problem and shows elevated RRs (ranging from 1.6 to 2.1) for all three upper quartiles versus the lowest quartile of cumulative exposure, although these data are limited by small numbers, and there is no significant trend.

Other limitations include possible inaccuracies in disease validation and ascertainment, including under-ascertainment of cases that were not self-reported by participants, lack of validation of the absence of disease among those who did not report autoimmune disease outcomes, and the possible exclusion of cases that could not be verified because of a lack of consent or inability to obtain appropriate medical records. We also lacked data on other potential environmental toxicants in the serum of Mid-Ohio Valley residents that could have confounded associations with PFOA, and given the limited information on the causes of autoimmune diseases, we cannot rule out possible confounding by other unmeasured exposures or characteristics.

Another limitation is that exposures were classified according to estimated historical serum levels based on predicted water concentrations and predicted air concentrations using an environmental fate and transport model, individual residential histories, and maps of public water supply networks, coupled with a one-compartment absorption and excretion model (Shin et al. 2011b). Model-estimated levels were correlated with measured levels in 2005–2006 (*r*_S_ = 0.67), based on 45,000 participants.

There are also a number of strengths to our analysis. We studied a well-defined cohort with documented high exposure to a chemical that has been reported to suppress the immune system in animals. Disease outcomes were confirmed through medical records review, with relatively large numbers for many autoimmune diseases. Nonetheless, our finding of a positive association between cumulative serum PFOA concentration and incident ulcerative colitis, and a lack of associations with other autoimmune diseases, are based only on one study and need replication.
